# Inhaled Gases for Neuroprotection of Neonates: A Review

**DOI:** 10.3389/fped.2019.00558

**Published:** 2020-01-27

**Authors:** Youness Tolaymat, Sylvain Doré, Hudson W. Griffin, Susana Shih, Mary E. Edwards, Michael D. Weiss

**Affiliations:** ^1^Department of Pediatrics, University of Florida, Gainesville, FL, United States; ^2^Departments of Neurology, Psychiatry, Pharmaceuticals and Neuroscience, University of Florida, Gainesville, FL, United States; ^3^Department of Anesthesiology, University of Florida, Gainesville, FL, United States; ^4^Health Science Center Libraries, University of Florida, Gainesville, FL, United States

**Keywords:** HIE, hypoxia-ischemia, inhaled gases, neuroprotection, neonatal

## Abstract

**Importance:** Hypoxic-ischemic encephalopathy (HIE) is a significant cause of morbidity and mortality in neonates. The incidence of HIE is 1–8 per 1,000 live births in developed countries. Whole-body hypothermia reduces the risk of disability or death, but 7 infants needed to be treated to prevent death or major neurodevelopmental disability. Inhalational gases may be promising synergistic agents due to their rapid onset and easy titratability.

**Objective:** To review current data on different inhaled gases with neuroprotective properties that may serve as adjunct therapies to hypothermia.

**Evidence review:** Literature review was performed using the PubMed database, google scholar, and ClinicalTrials.Gov. Results focused on articles published from January 1, 2005, through December 31, 2017. Articles published earlier than 2005 were included when appropriate for historical perspective. Our review emphasized preclinical and clinical studies relevant to the use of inhaled agents for neuroprotection.

**Findings:** Based on the relevance to our topic, 111 articles were selected pertaining to the incidence of HIE, pathophysiology of HIE, therapeutic hypothermia, and emerging therapies for hypoxic-ischemic encephalopathy in preclinical and clinical settings. Supplemental tables summarizes highly relevant 49 publications that were included in this review. The selected publications emphasize the emergence of promising inhaled gases that may improve neurologic survival and alleviate neurodevelopmental disability when combined with therapeutic hypothermia in the future.

**Conclusions:** Many inhaled agents have neuroprotective properties and could serve as an adjunct therapy to whole-body hypothermia. Inhaled agents are ideal due to their easy administration, titrability, and rapid onset and offset.

## Introduction

Hypoxic-ischemic encephalopathy (HIE) is a significant cause of morbidity and mortality in neonates. The incidence of HIE ranges from 1 to 8 per 1,000 live births in developed countries as high as 26 per 1,000 live births in underdeveloped countries (1).

Whole-body hypothermia reduces the risk of death or disability in infants with moderate or severe HIE (2–4). A metanalysis of 11 cooling trials in neonates with moderate to severe HIE demonstrated that to save 1 infant from either death or major neurodevelopmental disability, 7 infants must be cooled (5). In 2014, a multicenter randomized clinical trial evaluated increasing the depth of hypothermia to 32.0°C and the duration to 120 h, a change from original trials and the current clinical regimen of cooling to 33.5°C for 72 h. In this study infants were randomized to four hypothermia groups: 33.5°C for 72 h, 33.5°C for 120 h, 32°C for 72 h, or 32°C for 120 h. However, the study was terminated early due to concern of increased mortality that was higher in infants who underwent longer and deeper cooling (6). Of the children who were enrolled and were followed-up at 18–22 months of age, the primary outcome of death or severe neurodevelopmental disability at 18–22 months was 29.3% at 33.5°C for 72 h, 34.5% at 32.0°C for 72 h, 34.4%at 33.5°C for 120 h, and 28.2% at 32.0°C for 120 h (7). Therefore, we should strive for additional adjunct therapies to hypothermia that improve neurodevelopmental outcomes. Inhalational gases may serve as an ideal synergistic agent with hypothermia for neuroprotection due to their rapid onset and offset and titratability. This review will examine several promising inhalational gases that may offer synergistic neuroprotection when combined with hypothermia.

Currently, medical gases are commonly used in the medical field. Nitrous oxide and xenon are used as anesthetic agents, nitric oxide is used to treat pulmonary hypertension in neonates and adults and heliox is used in patients with upper and lower airway obstruction. The use of medical gases may be limited by cost. Nitric oxide treatment currently costs more than $100 per h, while xenon can costs $300 per h due to its rarity in atmospheric air (8, 9).

The use of inhaled gases as neuroprotective adjuncts with hypothermia warrants further evaluation of gas stability and effect on bioavailability and pharmacokinetics. While most reviewed studies reviewed in this article looked at inhaled gases in normothermic conditions, some animal studies have evaluated the use of inhaled gases in the setting of hypothermia (10–14). Furthermore, TOBY-Xe trial assessed the use of inhaled xenon as an adjunct therapy to therapeutic hypothermia (15).

## Pathophysiology of HIE

The mechanism of injury during hypoxia-ischemia (HI) provides a foundation for understanding the neuroprotective mechanisms of various inhalational gases. Oxygen and glucose are delivered to the brain in fetal life by cerebral blood flow. Cerebral blood flow helps the fetal brain maintain homeostasis and meet cellular energy demands. Acute sentinel events including placental abruption, prolapse of the umbilical cord, and uterine rupture decrease placental perfusion or disrupt the delivery of oxygen and glucose in the umbilical cord. Poor oxygenation from the placenta produces fetal hypoxia, which eventually leads to a decrease in fetal cardiac output. Subsequently, decreased fetal cardiac output reduces cerebral blood flow. Decreased cerebral perfusion sets in motion a temporal sequence of injury, which has distinct phases. In the acute phase, interruption of cerebral blood flow diminishes the delivery of oxygen and glucose to the fetal brain switching the cellular metabolism to anerobic glycolysis. Consequently, diminished ATP production leads to disturbances in intracellular sodium, water, and calcium as a result of dysfunctional transcellular transport (16). During depolarization, the excitatory amino acid glutamate, is released from presynaptic neurons. During hypoxia-ischemia there is an increased release of glutamate that leads to overstimulation of the glutamate receptors [i.e., α-amino-3-hydroxy-5-methyl-4-isoxazole propionate (AMPA), kainite (KA), and N-methyl-D-aspartate (NMDA)] on the postsynaptic neuron. Excitotoxicity results from overstimulation of glutamate receptors. Overstimulation of the KA and AMPA receptors causes sodium and chloride influx, leading to cellular hyperosmolality. Thus, water influx results in subcellular edema, which if severe enough can cause cell lysis. Overstimulation of the NMDA receptor triggers an influx of calcium. Calcium is the predominant second messenger in the cell. When calcium is present in pathological amounts, a series of enzymes are activated that destroys the cell. Calcium also contributes to the production of free radicals such as nitric oxide (NO), Fatty acid, and prostaglandin metabolism also generate free radicals, which lead to the formation of harmful amounts of superoxide and hydrogen peroxide. This cascade of events perpetuates injury through excitotoxicity. Free fatty acid peroxidation mediated by oxygen radicals causes worsening of the cellular injury (17). The culmination of energy failure, acidosis, glutamate mediated excitotoxicity, lipid peroxidation, and NO toxicity causes necrosis and apoptosis of cells in the brain (17).

A partial recovery ensues over the 30–60 min after resuscitation following the primary phase of injury. This partial recovery leads to a latent phase of injury (18). The latent phase may persist from 1 to 6 h and is characterized by recovery of oxidative metabolism, inflammation, and continuation of the activated apoptotic cascades (16). A secondary deterioration follows the latent phase in neonates with moderate to severe injury. The secondary phase of injury occurs within ~6–15 h after the injury. Cytotoxic edema, excitotoxicity, and “secondary energy failure” with nearly complete failure of mitochondrial activity characterize this second phase. Seizures typically occur in this secondary phase (19). A tertiary phase occurs over months after the acute insult and involves late cell death, remodeling of the injured brain, and astrogliosis (20).

Therapeutic hypothermia interrupts the pathophysiologic cascade of HIE during the acute phase of injury by limiting apoptosis and allowing time for recovery from oxidative stress. Proinflammatory pathway involved in the pathophysiology of HIE and characterized by production of cytokines such as IL-6, IL-8, and platelet-activating factor (PAF) are halted by 72 h of hypothermia (21, 22) Furthermore, excitotoxicity caused by influx of calcium is decreased by hypothermia (23). Hypothermia, in rats models of HI, prevents injury to the mitochondria by suppressing cytochrome C during the acute phase of injury (24).

## Evidence Review

We performed a literature review using the PubMed database, google scholar, and ClinicalTrials.Gov. The results focused on articles published between 2005 and 2017. Articles published prior to 2005 were incorporated when appropriate for historical perspective. Our review highlighted relevant preclinical and clinical studies that examined the use of inhaled agents for neuroprotection. Due to the overlap between adult stroke and neonatal HI, although the pathophysiology in HI tends to be more global, adult stroke studies were included because the studies provide potential information on therapies that may apply to neonatal HI and offer potential clues as to the mechanism of action of inhaled gases in neonatal HI. Therefore, we have included information from adult animal studies because neonatal studies are limited to broaden the perspective of each gas and the use as a potential therapy in neonatal HI.

In this article, we will discuss the emerging medical gases that have been evaluated in preclinical and clinical trials for neuroprotection in neonatal hypoxia-ischemia. Although many articles exist, it is beyond the scope of this review to focus on all articles related to the presented inhalational gases and every inhalational gas in the literature. To the make the review informative but concise, we have tried to select articles that provide representative information on the potential for each gas for neuroprotection in the preclinical and clinical setting and that highlight the potential mechanism of neuroprotection. We have selected inhalational gases that have demonstrated promise for potential translational applications in neonates with HIE. The text is augmented by Tables 1, 2 that briefly describe other articles from the literature that are not covered in the text.

**Table 1:** Summary of preclinical studies of inhaled gases in rodents.

**References**	**Gas**	**Age species**	**Procedure**	**Intervention**	**Outcomes**	**Behavioral**
David et al. (25)	Ar	Adult male rats	a) *In vitro*: neuronal cell slices were subjected to OGD b) *In vivo*: injection of NMDA into right striatum c) *In vivo*: MCAO transient ischemia for 1 h	a) OGD: Reperfusion and exposure to 25, 37.5, 50, or 75% Ar b) NMDA: 1 h after injection of NMDA rats were treated with either medical air (control), or 15, 25, 37.5, 50, or 75% Ar balanced with O_2_ or N_2_ c) 3 h post MCAO, rats were treated with either medical air (control) or 50% Ar	a) Less LDH production in Ar group b) Rats treated with 37.5 and 50% Ar had a significant reduction of neuronal death compared to NMDA control animals treated with medical air c) In MCAO, maximal neuroprotection by post-insult 50% Ar. When given 1 h after reperfusion, Ar reduced cortical volumes of brain damage but increased subcortical brain damage compared to control rats treated with air	Behavioral outcome assessed only for MCAO study rats. Rats treated with 50%Ar did not show improvement of neurologic deficits compared to control rats treated with medical air
Loetscher et al. (26)	Ar	Neonatal mice	Hippocampal slice cultures subjected to either OGD or a focal mechanical trauma	25, 50, or 74% Ar immediately after trauma or with a 2 or 3 h delay	a) Ar is neuroprotective in both O_2_ deprivation and mechanical brain trauma even when therapy is delayed for 3 h b) No difference in O_2_ deprivation between Ar concentrations while 50% Ar had best outcomes in mechanical brain trauma	N/A
Ryang et al. (27)	Ar	Adult rats	MCAO 2 h	Two groups:a) 50% Ar + 50% O_2_ (Ar group) b) 50% N_2_ + 50% O_2_ (control group) 1 h via face mask	Ar group had a significant reduction in the overall, cortical, and basal ganglia infarct volumes. Ar treatment resulted in a significant improvement of the composite adverse outcome	N/A
Zhao et al. (10)	Ar	Neonatal rats	a) *In vitro*: Cortical neuronal cell cultures challenged by OGD for 90 min b) *In vivo*: Rice-Vannucci HI model	a) Following OGD, cortical cells exposed to 70% Ar or N_2_ with 5% CO_2_ balanced with O_2_ at 33°C for 2 h b) Following HI: neonatal rats exposed to 70% Ar or N_2_ balanced with O_2_ at 33, 35, and 37°C for 2 h. Cortical and hippocampal infarction size was assessed at 4 weeks after treatment	a) Ar-HT increased phospho-Akt and HO1 expression and reduced the Tyr216-GSK-3β expression, cytochrome C release, and cell death in OGD-exposed cortical neurons b) Ar–HT treatment decreased infarct size and activated both caspase-3 and NF-κB in the cortex and hippocampus c) Ar-HT reduced hippocampal astrocyte activation and proliferation d) Ar-HT inhibited the PI3K/Akt pathway through LY294002 attenuated cerebral protection	N/A
Zhuang et al. (28)	Ar, He, Xe	Neonatal rats	Rice-Vannucci HI model	2 h after HI insult, animals were randomly exposed 90 min to:a) Ar, He, Xe (70% noble gas balanced with O_2_), or b) N_2_ (control group, 70% N_2_ balanced with O_2_)	Ar improved cell survival to naïve levels, whereas Xe and He did not. When tested against more severe HI injury only, Ar and Xe reduced infarct volume. Ar, He, and Xe increased the expression of Bcl-2, whereas He and Xe increased Bcl-xL. In addition, Bax expression was enhanced in the control and He groups	N/A
Cheng et al. (29)	CO	Neonatal mice	None	For 3 h:a) 0 ppm CO (air), b) 5 ppm CO in air, or c) 100 ppm CO in air	a) 3 h exposure to 5 or 100 ppm CO impaired cytochrome-c release, caspase-3 activation, and apoptosis in the neocortex and hippocampus b) CO increased NeuN protein and neuronal numbers and resulted in megalencephaly	4–5 weeks post exposure, mice underwent Morris water maze, and social approach-avoidance assay. Escape latency was significantly longer on day 1 and 2 of testing in both CO-exposed cohorts compared to controls. Air-exposed controls showed a marked increase in approach- avoidance score when the stimulus mouse was placed in the chamber
Queiroga et al. (30)	CO	Adult and neonatal rats	Apoptosis of astrocytes induced by diamide	Preconditioning with CO prior to apoptosis induction	CO prevented membrane depolarization induced by calcium and inhibited mitochondrial swelling. CO prevented membrane pore formation by increasing ANT activity	N/A
Queiroga et al. (31)	CO	Neonatal rats	Rice-Vannucci HI model	a) Sham surgery without hypoxia exposure (Control group) b) CO exposure prior to Sham surgery without hypoxia exposure (CO+Sham group) c) HI group, d) CO exposure prior to HI, (CO+HI group)	a) CO decreased apoptosis and increased Bcl-2 mRNA in primary cultures of neurons b) CO decreased apoptosis in the hippocampus, limited cytochrome-c released from mitochondria and reduced activation of caspase-3	N/A
Wang et al. (32)	CO	Adult male mice	Permanent MCAO	Administered for 18 h immediately after permanent MCAO:a) 250 ppm CO b) control air	a) Less brain damage than controls at 7 days b) 18 h CO treatment led to NRF2 dissociation from Keap1, nuclear translocation, increased binding activity of NRF2 to HO1 ARE, and elevated HO1 expression 6–48 h after CO exposure c) Loss of CO neuroprotection in NRF2 knock-out mice	N/A
Zeynalov and Doré (33)	CO	Adult mice	90 min MCAO	a) 125 ppm normal air or 250 ppm CO at onset of reperfusion b) 250 ppm CO inhalation 1 and 3 h after reperfusion	CO inhalation reduced infarct size and decreased brain edema	Improved neurological deficit scores at 48 h of survival time after ischemia
Liu et al. (34)	CO	Neonatal rats	Rice-Vannucci HI model	Eight groups:a) Sham b) Sham + electroacupuncture c) HIE d) HIE + electroacupuncture e) HIE + SAM f) HIBD + SAM + electroacupuncture g) HIE + HA h) HIE + HA + electroacupuncture	a) Electroacupuncture significantly downregulated the expression of nNOS and NF-κB in the rat cortex cells and alleviated cortical atrophy caused by HIE b) Increased intrinsic CO levels via overexpression of hemoxygenase-1 (HO1) in the cortex and was associated with alleviated cortical damage	N/A
Kohzuki et al. (35)	CO_2_	Neonatal rats	Rice-Vannucci HI model	6% CO_2_ delivered during HI	Smaller brain infarct size in rats exposed to CO_2_	Staircase test at 3 months of age showed improved forelimb strength in rats exposed to CO_2_
Vannucc et al. (36)	CO_2_	Neonatal rats	Rice-Vannucci HI model	a) 0% (hypocapnia) b) 3% (normocapnia) c) 6% CO_2_ (hypercapnia), all with 8% O_2_, balanced with N_2_	a) Hypocapnia was associated with decreased cerebral blood flow b) Normocapnia and hypercapnia groups showed preservation of cerebral blood flow in addition to maintaining higher cerebral glucose and lower lactate concentrations	N/A
Vannucc et al. (37)	CO_2_	Neonatal rats	Rice-Vannucci HI model	3, 12, or 15% CO_2_ for 2 h at 37°C	a) Inhaled 12 and 15% CO_2_ was associated with blood CO_2_ tension of 80 and 100 mmHg, respectively b) Extreme hypercapnia (15% CO_2_) was associated with severe brain infarcts following HI insult and significant reduction of cerebral blood flow when compared to the other groups	N/A
Vannucc et al. (38)	CO_2_	Neonatal rats	Rice-Vannucci HI model	0, 3, 6, or 9% CO_2_	a) Mild hypercapnia (6% CO_2_) had the lowest brain atrophy followed by normocapnia (3% CO_2_), while hypocapnia (0% CO_2_) had the worst damage b) CO_2_ exposure was associated with lower lactate levels and improved glucose concentrations by preventing hypoglycemia	N/A
Cai et al. (39)	H_2_	Neonatal rats	Rice-Vannucci HI model	Neonatal rats: Intra-ischemic treatment and post-ischemic treatmenta) Sham b) HI c) HI+ H_2_ Adult rats: a) MCAO b) MCAO+ H_2_,	H_2_ treatment significantly reduced the number of positive TUNEL cells and suppressed caspase-3 and−12 activities	N/A
Matchett et al. (40)	H_2_	Neonatal and adult rats	Neonatal rats: Modified Rice-Vannucci HI model 120 and 150 min Adult rats: MCAO	Neonatal HI:a) Sham b) Sham + intra-ischemia H_2_ × 2 h c) HI only d) HI+ Preconditioning with H_2_ × 1.5 h e) HI+ post-ischemia H_2_ × 1 h Adult MCAO: a) Sham b) HI only c) HI+ intra-ischemia H_2_ × 2.5 h d) HI+ post-ischemia H_2_ × 1 h e) HI + post-operative-ischemia x 1.5 h	a) H_2_ therapy in neonatal HI was not associated with decreased volume of infarction or decreased concentration of MDA b) H_2_ pretreatment was associated with increased infarction volume in neonatal HI c) Exposure of H_2_ to non-ischemic neonates was associated with a significant increase in brain concentration of MDA	N/A
Ohsawa et al. (41)	H_2_	Adult rats	*In vitro*: neuronal cell culture treated with a) antimycin A and menadione b) OGD *In vivo*: Adult male rats underwent MCAO x 90 min followed by reperfusion x 30 min	Four groups:a) 1% H_2_/30% O_2_/69% N_2_O b) 2% H_2_/30% O_2_/68% N_2_O c) 4% H_2_/30% O_2_/66% N_2_O 0% H_2_/30% O_2_/70% N_2_O H_2_ was administered either for the entire procedure (120 min), last 35 min, or first 85 min	a) Decreased oxidative stress and infarction b) Reduction of cytotoxic radicals c) Induced cytoprotective factors that prolong cell life	N/A
Li et al. (42)	He	Neonatal rats	Rice-Vannucci HI model	a) Normal control group b) He-Preconditioning group (70% He/30% O_2_ for three 5-min periods) c) HIE group d) He-Preconditioning + HIE group e) L-NAME + HIE group f) L- NAME + He-Preconditioning + HIE group	a) Reduction in infarct size and increase in NO content in the brain b) Increased anti-oxidase expression and DNA binding activity of NRF-2. Suggests that preconditioning promotes intrinsic NO production that is neuroprotective	Tested at 3 weeks. He-preconditioning group had better scores in the wire hang and beam balance tests compared to the HIE group.
Pan et al. (43)	He	Adult male rats	MCAO for 2 h and a 1 h reperfusion	During stroke and reperfusion rats were subjected to either:a) 30% O_2_/70% N_2_ (control group) b) 100% O_2_ (hyperoxia group), OR c) 30% O_2_ /70% He (heliox group)	a) Infarct volume in the heliox group was smaller than the hyperoxia and control groups b) Neurologic scores in the heliox group were significantly better compared with controls	Neurologic scores 1 and 24 h post MCAO in the heliox group were significantly better compared with controls using Hunter neurological scores
Li et al. (44)	NO	Adult mice	Transient MCAO for 1 h followed by reperfusion for 47 h	Inhaled NO at 10, 20, 40, 60, or 80 ppm. Each group was subdivided into four duration groups for 5, 8, 16, or 24 h beginning immediately after MCAO	a) At 10 ppm only the 24 h duration group exhibited reduced infarct volume b) At 20, 40, and 60 ppm, only 8 and 16 h of exposure led to smaller infarctions c) 60 ppm iNO could be transported from lung to brain d) iNO administered for 8 h improved recovery from subarachnoid hemorrhage and reduced the inflammatory response accompanying ischemic stroke	N/A
Lu et al. (45)	NO	Neonatal rats	a) Neuronal cells underwent OGD × 90 min b) Modified Rice-Vannucci HI model	No intervention	a) OGD increased NO generation b) HA disrupted iron regulation c) Hydroxyl radicals and iron deposition were ameliorated by NOS- inhibited aconitase activity and lead to cellular iron accumulation	N/A
Terpolilli et al. (46)	NO	Adult male rats, Adult male mice, and Neonatal mice	a) Adult rats underwent MCAO b) Adult mice underwent bilateral carotid banding c) Neonatal mice ligature of the left common carotid artery, 1 h later, hypoxia for 50 min with or without addition of 50 ppm NO to the gas mixture	50 ppm NO	a) In adult mice, inhaled NO enhanced blood flow during reperfusion and reduced inflammation b) Pups that received iNO at the time of hypoxic injury had smaller infarct volume and lower histopathological scores compared to controls	N/A
Zhu et al. (47)	NO	Neonatal mice	Rice-Vannucci HI model	50 ppm NO mixed with N_2_ vs. N_2_ alone for 50 min	Reduced brain injury and tissue loss. Only male mice had significant reduction of infarct size	N/A
Calvert et al. (48)	O_2_	Neonatal rats	Rice-Vannucci HI model	Three groups:a) HI b) HI and HBO; 100% O_2_ at 3 ATA for 1 h (following recovery for 1 h) c) Control (no anesthesia, carotid ligation, hypoxia, or HBO exposure)	HBO decreased caspase-3 activity, PARP cleavage and DNA fragmentation. HBO preserved brain weight.	N/A
Dalen et al. (49)	O_2_	Neonatal rats	Rice-Vannucci HA model	HI followed by 30 min reoxygenation in 21% O_2_ or 100% O_2_ before 5 h of NT (37°C) or HT (32°C)	Reoxygenation with 100% O_2_ increased hippocampal injury score and negated HT neuroprotection	Reoxygenation with 100% O_2_ worsened reflex performance in staircase test
Smit et al. (50)	O_2_	Neonatal rats	Modified Rice-Vannucci HI model	Soon after HI, pups underwent immediate resuscitation in either 21 or 100% O_2_ for 30 min	No significant change in brain atrophy	No change in short-term neurologic outcome
Dingley et al. (51)	Xe	Neonatal rats	Rice-Vannucci HI model	After HI insult, 3 h inhalation ofa) 50% Xe/30% O_2_/20% N_2_ b) 30% O_2_/70% N_2_	a) One week after HI survival, significant global protection in the Xe group (80% less injury) b) Percentage of global damage score in non-Xe vs. Xe groups: cortex/white matter (88 vs. 25%); hippocampus (62 vs. 0%); basal ganglia (81 vs. 25%); and thalamus (50 vs. 0%), respectively	N/A
Hobbs et al. (12)	Xe	Neonatal rats	Rice-Vannucci HI model	a) NT 37°C b) HT32°C c) Xe 50% + NT37°C d) Xe 50% + HT32°C	a) Xe 50% + HT32°C produced the greatest improvement (71%) in global histopathology scores. The overall effect of HT and Xe is additive) Xe 50% and HT32°C individually produced smaller improvements	Staircase testing (long-term) was performed from 8 to 11 weeks. Results: a) Xe 50% + HT32°C group: complete restoration of long-term functional outcomes b) Untreated pups (NT37°C) reached an early performance plateau of staircase test c) The Xe 50%+NT37°C or HT32°C only groups, had mild improvement in staircase testing
Luo et al. (52)	Xe	Neonatal mice	a) *In vitro*: OGD 1 h after preconditioning of cells for 2 h b) *In vivo*: 4 h after preconditioning for 120 min, mice underwent Rice-Vannucci HI model	*In vitro:* Neuronal cells were preconditioned for 120 min with 12.5, 25, 50, or 75% Xe, 0.67–3.3% sevoflurane, or a combination of both *In vivo:* Neonatal mice were preconditioned with 20 or 75% Xe, 0.75 or 1.5% sevoflurane, or 20% Xe plus 0.75% sevoflurane in 25% O_2_ balanced with N_2_	a) *In vitro:* Preconditioning with Xe for 2 h produces a concentration-dependent reduction in LDH release after OGD. LDH release was significantly reduced by Xe concentrations of 50 and 75% b) *In vivo:* Although sevoflurane alone did not reduce infarct size in Rice-Vannucci model of neonatal HI injury, Xe alone or in combination with sevoflurane reduced infarct size and improved neuromotor function at 30 days with slight advantage of the second group	N/A
Ma et al. (53)	Xe	Adult mice	24 h after preconditioning: a) bilateral renal pedicle clamping for 25 min or b) right renal pedicle clamping for 40 min and left nephrectomy c) Sham-operated mice had dissection as above but with no occlusion of the renal vessel	Preconditioning for 3 h with 70% Xe, 70% N_2_, or 70% N_2_O balanced with 30% O_2_ for 2 h or 8% O_2_ balanced with N_2_	Xe preconditioning protects against intermittent renal ischemia through increased expression of HIF-1α Knockout mice lost protective effect	N/A
Ma et al. (54)	Xe	Neonatal rats	*In vitro*: cell culture OGD *In vivo*: Rice-Vannucci HA model	*In vitro*: Neuronal culture cells were preconditioned with Xe for 2 h. *In vivo*:a) Treatment group was preconditioned with 70% Xe+30% O_2_ for 120 min b) Control group was exposed to 70% N_2_O balanced with O_2_ for 120 min	a) Concentration-dependent reduction of LDH release from cells with OGD 24 h later b) Preconditioning with Xe decreased propidium iodide staining in a hippocampal slice culture model subjected to OGD c) Preconditioning with Xe reduced infarction size when assessed 7 days after injury d) pCREB was increased by Xe exposure	N/A
Martin et al. (13)	Xe	Neonatal rats	Rice-Vannucci HI model	After a 1 h recovery period, rats received asynchronous administration ofa) Mild HT (35.8°C) and 20% Xe with a 1- or 5-h gap between interventions b) 20% Xe alone c) Mild HT (35.8°C) alone	a) Brain infarct was reduced by Xe and HT administration at 1 h intervals after 90 min of HI b) Administration of 20% Xe after 6 h of HI and 2-h administration of 35.8°C HT 1 h following the HI insult was not neuroprotective	N/A
Sabir et al. (14)	Xe	Neonatal rats	Rice-Vannucci HI model	a) Immediate NT-37°C for 5 h b) Immediate HT-32°C: Trectal 32°C for 5 h c) Immediate HT-32°C plus 50% inhaled Xe for 5 h	a) No difference in neuronal cell count in the subventricular zone among different treatment groups b) No reduction in brain area loss in either the HT group or in the HT plus 50% inhaled Xe group	N/A
Sabir et al. (55)	Xe	Neonatal rats	Rice-Vannucci HI model	a) ${\text{NT}}_{37^{{}^{\circ}}\text{C}}$, ${\text{HT}}_{35^{{}^{\circ}}\text{C}}$, or ${\text{HT}}_{32^{{}^{\circ}}\text{C}}$ or Xe concentrations (0, 20, or 50%) starting immediately or with a 4-h delay b) P7 pups were exposed to ${\text{HT}}_{35^{{}^{\circ}}\text{C}}$, NT+Xe_20%_ or ${\text{HT}}_{35^{{}^{\circ}}\text{C}}$+Xe_20%_ starting with a 4-h delay after the insult	a) Immediate HT to 32°C but not 35°C significantly reduced infarct size b) Synergistic effect when Xe immediately added to ${\text{HT}}_{32^{{}^{\circ}}\text{C}}$	N/A
Thoresen et al. (11)	Xe	Neonatal rats	Rice-Vannucci HA model	a) 1 h of 50% Xe during the first h of HT (1 h immediate Xe) plus 3 h HT b) 1 h of 50% Xe during the last h of HT (1 h delayed Xe) plus 3 h HT COMPARED TO c) 3 h of both 50% Xe and HT	a) Combination of Xe + HT is superior to HT alone with the best results in 3 h of both Xe and HT group b) Longer duration of Xe administration combined with HT was more effective (3 h better than 1 h of Xe) c) No difference between early or late administration of Xe (immediate vs. delayed)	Female rats showed better motor skills than males
Yang et al. (56)	Xe	Neonatal rats (*in utero*)	Fetal Asphyxia: Dams were sacrificed by cervical dislocation and uterine horns were removed and placed in a water bath at 37°C for 10 min to induce HI insult 4 h after gas exposure	Pregnant rats were preconditioned at day 22 of gestation with either 0.35% sevoflurane or 35% Xe from 9 a.m. until the pup was delivered	Compared to the control group, Xe, and sevoflurane preconditioned pups had lower caspase 3 levels and higher cell viability at prenatal days 3 and 7	Xe- and sevoflurane-preconditioned pups had improved cognitive testing with Morris water maze at postnatal day 50

**Table 2:** Summary of preclinical studies of inhaled gases in large animal models.

**References**	**Gas**	**Age species**	**Procedure**	**Intervention**	**Outcomes**	**Behavioral**
Alderliesten et al. (57)	Ar	Newborn piglets	Hypoxia by inhaling 8% O_2_ x1 h	a) Titration up from 30% Ar x1h then 50% Ar x1h to 80% Ar x1h b) Hypoxia+50% Ar x3 periods of 1 h c) Hypoxia+hypothermia+ 50% Ar x3 periods of 1 h d) Cage animal control	No hemodynamic instability in Ar group (HR, BP, cerebral oxygen saturation, or blood gas)	N/A
Broad et al. (58)	Ar	Newborn piglets	Occluding common carotid arteries bilaterally and reducing inspired FiO_2_ to 6%	a) Hypothermia (HT) to 33.5°C x24 h b) HT + 45–50% Ar x24 h	HT with argon preserved brain MRS (magnetic resonance spectroscopy) ATP, decreased MRS lactate/NAA peak, and expedited EEG background recovery when compared to HT alone	N/A
Oláh et al. (59)	H_2_	Newborn piglets	Asphyxia by clamping ET tube x8 min	a) Time control b) Asphyxia+RA (room air) c) Asphyxia+2.1%H_2_ x4 h	a) H_2_-treated animals showed improved EEG activity recovery b) H_2_ fully or partially preserved cerebrovascular reactivity and provided modest neuroprotection	N/A
Varga et al. (60)	H_2_	Newborn piglets	Asphyxia by tracheal occlusion x8 min followed by ventilation with (6% O_2_, 20% CO_2_ gas mixture x20 min	a) RA(room air) control group b) Asphyxia+RA x24 h c) Asphyxia+2.1% H_2_ x4 h then RA x20 h	H_2_ treatment prohibited increase in COX-2 build-up in neurons	N/A
Domoki et al. (61)	H_2_	Newborn piglets	Asphyxia: Clamping endotracheal tube in intubated piglets x10 min. Both Sham groups were ventilated in RA for 10 min. Following asphyxia, animals were ventilated with respective gases for 4 h then euthanized	a) Asphyxia/ventilation with RA (21% O_2_, 79% N_2_) ventilation b) Asphyxia/ventilation with H_2_-RA (2.1% H_2_; 21% O_2_; 76.9% N_2_) ventilation c) Time control group (Sham) ventilated with RA d) Time control group (Sham) ventilated with H_2_-RA	H_2_-RA ventilation: a) Reduced brain injury in all brain areas examined b) Improved cerebrovascular reactivity to hypercapnia but not to NMDA	N/A
Linner et al. (62)	O_2_	Newborn piglets	Asphyxia in newborn piglets (heart rate < 60 BMP, mean arterial pressure < 30 mmHg)	10 min resuscitation with either: a) 1 breath/min of 100% O_2_ b) 1 breath/min of air	Piglets in oxygen group did not require closed-chest cardiac massage (resuscitation) All piglets in room air group required closed-chest cardiac massage	N/A
Solas et al. (63)	O_2_	Newborn piglets	Hypoxia-ischemia-hypercapnia (HIH). Hypoxia: breathing 8% O_2_ in N_2_ Ischemia: temporary bilateral common carotid artery occlusion Hypercapnia: mixing CO_2_ with inspiratory gas targeting (PaCO_2_ 8–9 kPa)	Two groups: a) HIH 100%: HIH x20 min, reperfusion, re-oxygenation with 100% O_2_ x30 min followed by 21% O_2_ x90 min b) HIH 21%: HIH x20 min, reperfusion, re-oxygenation with 21% O_2_ x120 min	a) HIH 100% had improved restoration of mean arterial blood pressure b) No difference in amino acid build up in cerebral striatum (glutamate, taurine, and alanine) between both groups	N/A
Solberg et al. (64)	O_2_	Newborn piglets	Hypoxia by inhaling 8% O2 in N_2_ until either the base excess reaches ~20 mM or the mean arterial blood pressure > 15 mm Hg	Resuscitation x30 min with a) 21% O_2_ after hypoxia b) 40% O_2_ after hypoxia c) 100% O_2_ after hypoxia d) 100% O_2_ without hypoxia	Resuscitation with 100% O_2_ increased net matrix metalloproteinase gelatinolytic activity in corpus striatum, and caspase-3 expression and activity compared to those resuscitated in 21% O_2_	N/A
Kutzsche et al. (65)	O_2_	Newborn piglets	Hypoxia by inhaling 8% O_2_ x20 min	Reoxygenation x30 min with: a) 100% O_2_ b) 21% O_2_, subdivided to L-NAME vs. saline placebo	L-NAME pretreatment decreased cerebral perfusion and systemic blood pressure during hypoxemia. Reoxygenation with 100% O_2_ with no adverse effect on cerebral perfusion.	N/A
Kutzsche et al. (66)	O_2_	Newborn piglets	Hypoxia by inhaling 6% O_2_ x20 min	Reoxygenation x30 min with: a) 100% O_2_ b) 21% O_2_	a) Hypoxia decreased cerebral nitric oxide (NO) concentration and decreased cerebral perfusion b) Both groups had restoration of NO concentration with reperfusion. However, 100% O_2_ group had a higher level after reoxygenation	N/A
Chakkarapani et al. (67)	Xe	Newborn piglets	Hypoxia by inhaling 5–7% O_2_ until EEG background activity is suppressed to <7 uV	Three Xe treatment groups: a) 50%Xe, 30% O_2_, 20%N_2_ + normothermia 38.5°C—normal for pigs (NT) b) 50%Xe, 30% O_2_, 20%N_2_ + hypothermia 33.5°C (HT) x12H c) 50%Xe, 30% O_2_, 20%N_2_ + hypothermia 33.5°C (HT) x24H	a) Xe had no effect on heart rate b) Xe improved mean arterial blood pressure c) No negative hemodynamic interaction between Xe and hypothermia	N/A
Faulkner et al. (68)	Xe	Newborn piglets	Occlusion of common carotid arteries bilaterally and reducing inspired O_2_ to 12% x10 min	Four groups: a) Normothermia (NT) b) NT + 50%Xe x24H c) Hypothermia 33.5 C (HT) x24H d) HT + 50%Xe x24H	a) Mean arterial blood pressure is lower in all groups compared to NT b) HTx24H and HT+ 50% Xe x24H both decreased cerebral magnetic resonance spectroscopy abnormalities, nuclear DNA fragmentation using TUNEL staining, and caspase 3 activity in the cortex c) Added Xe to HT did not reach statistical significance of neuroprotection when compared to HT alone	N/A

*ANT, adenine nucleotide translocase; Ar, argon; ARE, antioxidant response elements; ATA, atmospheres absolute; Bax, Bcl2-associated X; Bcl-2, B-cell lymphoma 2; Bcl-xL, B-cell lymphoma-extra-large; cAMP, cyclic adenosine 3′,5′-monophosphate; CO_2_, carbon dioxide; HA, hydroxylamine; HBO, hyperbaric oxygen; He, helium; HI, hypoxia-ischemia; HIBD, hypoxic-ischemic brain damage; HO-1, heme oxygenase-1; HT, hypothermia; Keap1, kelch-like ECH-associated protein 1; LDH, lactate dehydrogenase; L-NAME, N(ω)-nitro-L-arginine methyl ester; MCAO; middle cerebral artery occlusion; MDA, malondialdehyde; N/A, not applicable; N_2_, nitrogen gas; NF-κB, nuclear factor-κB; nNOS, neuronal nitric oxide synthase; N2O, nitrous oxide; NRF-_2_, nuclear factor erythroid 2-related factor 2; NT, normothermia; O_2_, oxygen gas; OGD, oxygen glucose deprivation; PARP, poly(ADP-ribose) polymerase; pCREB, phosphorylated cAMP response element binding protein; PI3K, phosphoinositide-3-kinase; RA, room air; SAM, S-adenosyl-L-methionine; TUNEL, terminal deoxynucleotidyl transferase dUTP nick end labeling; Tyr216-GSK-3β, Phospho-glycogen synthase kinase-3β Tyr-216; Xe, xenon*.

### Xenon

Xenon is one of the most studied inhaled gases. As a noble gas, it is not reactive under normal conditions. Xenon crosses the blood-brain barrier, has a fast onset of action, is an anesthetic at atmospheric pressure, and is safe in neonates undergoing hypothermia (51). Xenon is approved as an anesthetic drug. A multicenter international study showed that inhaled 65% xenon was not inferior to inhaled sevoflurane or intravenous propofol as an anesthetic agent in coronary bypass graft surgery. Furthermore, xenon administration was associated with less troponin-I release when compared to propofol (69). It is cardio- and nephro-protective and is not fetotoxic (53, 70, 71). Xenon's neuroprotective effects are mediated by antagonizing the NMDA receptor channel (72, 73). Xenon also blocks AMPA, which is a glutamate receptor (74). In addition, xenon interferes with the calcium/calmodulin-activated kinase II complex (75). Although xenon has properties that make it an ideal therapeutic gas, it is expensive and scarce in atmospheric air. Xenon has no effect on heart rate and improves blood pressure during hypothermia in a preclinical piglet study (67).

#### Preclinical Studies

Xenon is a potential neuroprotective agent following hypoxic-ischemic injury because it transiently blocks NMDA and AMPA receptors involved with excitotoxicity (Supplementary Figure 1). Neonatal rats that experienced left carotid artery ligation for 90 min were treated with 3 h of inhaled 50% xenon. Histopathological evaluation 1 week from the time of injury showed that rats treated with xenon had improved preservation of the cortex, hippocampus, basal ganglia, and thalamus (76).

Xenon preconditioning is also preventive against ischemia-induced neuronal injury. *In vitro*, pre-exposure to xenon before oxygen and glucose deprivation (OGD) reduces lactate dehydrogenase (LDH) and propidium iodide staining in neuronal cell cultures. Neonatal rats undergoing HI following preconditioning with 70% xenon had smaller infarct sizes and elevated levels of phosphorylated cAMP response element-binding protein (pCREB) and B-cell lymphoma 2 (Bcl-2) (54). Bcl-2 is a prosurvival protein that decreases apoptosis and is upregulated by pCREB (77).

Sevoflurane, a volatile anesthetic that blocks γ-aminobutyric acid type A receptors (GABA), synergistically augments the neuroprotective effects of xenon. *In vitro*, preconditioning with a combination of xenon and sevoflurane for 2 h before OGD had a greater reduction of LDH release. Although sevoflurane alone did not reduce infarct size in a Rice-Vannucci model of neonatal hypoxic-ischemic injury, xenon alone and in combination with sevoflurane reduced infarct size and improved neuromotor function at 30 days. The xenon-sevoflurane group had a slightly improved effect (52). In another study, pregnant rats were preconditioned at day 22 of gestation with 35% xenon or 0.35% sevoflurane before sacrificing dams. After sacrifice, uterine horns were placed in 37°C water for 10 min to simulate neonatal asphyxia. Compared to the control group, xenon- and sevoflurane-preconditioned pups had lower caspase-3 levels in the hippocampus and higher cell viability at postnatal days 3 and 7. These preconditioned pups also showed improved cognition in the Morris water maze test at postnatal day 50 (56).

Neonatal rats undergoing the Rice-Vannucci model of hypoxic-ischemic injury for 90 min received xenon at 1 and 5 h after initiation of hypothermia (32°C). These rats had significantly reduced infarct volume (13). A combined therapy of xenon and hypothermia was found to decrease infarct volume and improved short and long-term functional testing compared to each treatment alone (11, 12). However, a recent study with a larger sample size by the same group failed to replicate the results and did not show a synergistic effect (55). In another study, neonatal rats received xenon combined with hypothermia after undergoing hypoxia for 150 min due to common carotid ligation. These rats did not show altered hemispheric loss (14). Hypothermia augmented with xenon in asphyxiated newborn piglets had decreased markers of brain injury as measured by cerebral magnetic resonance spectroscopy, decreased nuclear DNA fragmentation using TUNEL staining and caspase 3 activity in the cortex. However, combining xenon with hypothermia was not superior to hypothermia alone (68).

#### Clinical Studies

A feasibility study combined xenon with hypothermia in a single-arm, dose-escalation design. The dose of Xenon was increased from 25 to 50%. The study did not find adverse respiratory or cardiovascular effects when xenon was combined with hypothermia (51). Administering 50% Xenon would limit fractional inspired oxygen delivery to infants to 50% which may restrict its clinical use in neonates with HIE that have hypoxemic respiratory failure or persistent pulmonary hypertension.

“Total Body hypothermia plus Xenon (TOBY-Xe)” was a randomized, open-label, parallel-group trial done in 4 Neonatal intensive care units in the UK (NCT 00934700). Within 12 h of birth, patients with moderate to severe HIE were randomized to either hypothermia (33.5°C) for 72 h or cooling combined with 30% inhaled xenon for 24 h. Two primary outcomes were measured within 15 days of birth. First, the lactate/NAA ratio in the thalamus was measured with magnetic resonance spectroscopy and was reduced. Unlike lactate which rises as a result of ischemia, N-acetyl aspartate (NAA) is an amino acid found naturally in the brain. Elevation of lactate/NAA is a marker of brain hypoxic-ischemic injury. Second, the fractional anisotropy in the posterior limb of the internal capsule was measured with MRI and was preserved. Similarly to the lactate/NAA ratio, fractional anisotropy is a marker of injury to the white matter tracts after hypoxic-ischemic injury. The study did not show statistical differences between the two groups in the lactate to NAA ratio in the thalamus or in the fractional anisotropy in the posterior limb of the internal capsule (15). Lack of effect of xenon may be related to the delayed administration of xenon to neonates with HIE at a median of 10 h of age. Additionally, the authors also reported that 30% xenon which is sub-anesthetic dosing (anesthetic dosing is achieved with alveolar xenon concentration of 60%) was effective in suppressing seizures associated with HIE, which is an effect that was not previously reported (78).

“Xenon and Cooling Therapy in Babies at High Risk of Brain Injury Following Poor Condition at Birth (CoolXenon3)” is an ongoing clinical trial evaluating the benefit of inhaled xenon gas treating newborn infants with HIE in combination with cooling (NCT02071394). The study is comparing 72 h of whole-body hypothermia starting within 3 h after birth vs. whole-body hypothermia in addition to 50% xenon inhalation via endotracheal tube for 18 h starting within 5 h after birth. The primary outcome of the study is death or moderate to severe disability at 18 months of age.

### Argon

The interest in studying other noble gases such as argon grew after xenon emerged as a neuroprotective agent. Unlike xenon, argon is more abundant and cheaper to manufacture, and it is not a sedative. Argon's mechanism of action for neuroprotection is not well-understood. The blockage of GABA receptors at high atmospheric pressure is a proposed mechanism of neuroprotection (79). Argon has no effect on heart rate, blood pressure, cerebral oxygen saturation, or blood gas in a preclinical piglet study (57).

#### Preclinical Studies

*In vitro*, argon exposure led to improved neuronal cell survival after OGD and mechanical trauma (26). David et al. (25). showed that neuronal cells exposed to argon following OGD produced less LDH. Adult rats that underwent either transient cerebral ischemia for 60 min by middle cerebral artery occlusion (MCAO) or intrastriatal injection of NMDA were assessed for subcortical and cortical damage. Argon inhalation 1 h after NMDA injection to the right striatum significantly reduced cortical infarction size compared to the contralateral region. The maximum effect of argon inhalation was noted at a volume of 50%. However, argon therapy after transient MCAO alleviated histopathologic cortical volume loss by 35% but increased subcortical volume loss by 35% (25). Adult rats treated with inhaled argon 1 h after transient MCAO demonstrated statistically significant reductions in infarct volumes at the levels of the cortex and basal ganglia without changes in the of mortality risk (27).

*In vitro*, exposure to 70% argon at 33°C for 2 h following OGD resulted in upregulation and subsequent increased expression of heme oxygenase-1 (HO-1), Bcl-2, and phospho-Akt in cortex and hypothalamus neuronal cell cultures (10). Conversely, phospho-glycogen synthase kinase-3β Tyr-216 (Tyr216-GSK-3β) expression was decreased. When the effects of HO-1 and phospho-Akt were blocked, argon's neuroprotective effect was lost at the cortical level, proving that neuroprotection was mediated by argon. *In vivo*, when Argon was combined with hypothermia for 2 h in neonatal rats following the Rice-Vannucci model of HI, the infarct volume and level of caspase-3 decreased. Although hypothermia alone had no positive effect on Nuclear Factor-kB (NF-κB), argon combined with hypothermia reduced the expression of NF-κB, which is a main inflammatory cascade component in the brain (10). In a piglet study argon combined with hypothermia preserved brain MRS (magnetic resonance spectroscopy) ATP, decreased MRS lactate/NAA peak, and expedited EEG background recovery when compared to hypothermia alone (58).

#### Clinical Trials

Currently, no one has performed a clinical trial of argon as a neuroprotective agent.

### Helium

Helium is odorless, colorless, and tasteless. Heliox, a combination of 79% helium and 21% oxygen, which has lower viscosity than medical air has been used widely in patients with upper airway obstruction, to decrease work of breathing (80). In addition, it attenuates lung inflammation following acute lung injury of neonatal piglets (81). Preconditioning with helium protects the heart myocardium against HI (82). Helium was not shown to change cerebral blood flow in a human study (83).

#### Preclinical Studies

Infarct volumes and neurologic scores were assessed in adult rats that underwent left MCAO for 2 h followed by reperfusion for 1 h. Adult male rats breathed 30% oxygen and 70% nitrogen (control group), 100% oxygen (hyperoxia group), or 30% oxygen and 70% helium (heliox group). The infarct volume in the heliox group was smaller than the hyperoxia and control groups. Neurologic scores in the heliox group were significantly better compared to the control group (43). Neonatal pups preconditioned with helium 24 h prior to Rice-Vannucci model of HI for three 5-min periods demonstrated increased endogenous of nitric oxide production, increased the transcriptional factor Nrf2 activation and anti-oxidant enzyme expression, reduced brain infarct areas, and improved neurological testing scores when tested 2 weeks following injury. This suggests that the mechanism of neuroprotection of helium preconditioning is mediated by activation of antioxidase response element by NO (Supplementary Figure 1) (42). Similarly, inhaled helium (70% balanced with oxygen for 90 min) administered to neonatal rats following the Rice-Vannucci HI model increased neuronal cell survival but did not decrease infarct volume in prolonged HI (HI for 120 vs. 90 min) (28).

#### Clinical Trials

No one has performed a clinical study with helium as a neuroprotective agent.

### Hydrogen

Hydrogen is both an odorless and colorless gas. It has the lowest density of all gases and is by far the most abundant element in the universe, found on most stars and planets. It is primarily bound to oxygen as water with very scarce amounts in atmospheric air. Hydrogen is thought to protect against HI injury by binding free radicals formed by oxidative stress (i.e., hydroxyl radicals) (41, 84) and reducing cyclooxygenase-2 (COX-2) accumulation in the cerebral cortex (60).

#### Preclinical Studies

Adult mice that consumed hydrogen water had reduced oxidative stress in the hippocampus caused by chronic physical restraint (85). Inhaled hydrogen was also found to protect against intestinal (86) and myocardial ischemia in rats (87).

Hydrogen treatment for 30, 60, and 120 min decreased infarct size and brain apoptosis by reducing caspase-3 and caspase-12 activity in neonatal rats undergoing the Rice-Vannucci model of HI for 90 min (Supplementary Figure 1) (39). In a similar study that extended HI to 120 and 150 min, neuroprotection was not shown (40). Furthermore, the latter study reported an increased infarct volume when rats were preconditioned with hydrogen more than 90 min prior to HI.

Newborn piglets subjected to asphyxia for 10 min followed by re-ventilation with hydrogen supplemented room air were shown to have ameliorated neuronal injury in the cortex, hippocampus, basal ganglia, cerebellum, and brain stem (61). Another study of newborn piglets treated with inhaled 2.1% H_2_ after asphyxia by clamping the endotracheal tube for 8 min showed improved EEG activity recovery and preservation of cerebrovascular reactivity (59).

#### Clinical Trials

Currently hydrogen is not used for neuroprotection in ongoing or completed clinical studies.

### Nitric Oxide

NO produced by endothelial cells and acts as a potent vasodilator that is rapidly metabolized by oxyhemoglobin. Clinicians manage persistent pulmonary hypertension in neonates with inhaled NO (iNO) because it vasodilates the pulmonary vascular bed. Recent research showed that endogenous cardiac NO binds to plasma proteins and can affect distant organs such as the liver. This finding indicates that NO may have a broad effect in the human body (88). Inhaled nitric oxide had no effect on hemodynamics in rats treated with hypothermia following prolonged cardiopulmonary arrest (89).

#### Preclinical Studies

*In vitro*, OGD increases iron deposition and hydroxyl radical formation in neonatal brain hippocampal cell cultures. This process is mediated by endogenous production of NO. Neuronal cellular death is reduced by scavenging iron with deferoxamine and by blocking endogenous NO production by using L-NAME [N(ω)-nitro-L-arginine methyl ester] (45).

On the contrary, exogenous administration of 50 ppm iNO administered immediately after transient MCAO of adult mice was associated with selective dilation of arterioles in the ischemic areas. This dilation exclusively improved collateral blood flow and significantly reduced infarct size. Treatment with iNO for 24 h enhanced long-term adverse neurologic outcomes and survival in mice. This same study looked at perinatal asphyxia. Following the ligature of the left common carotid artery, neonatal pups were subjected to hypoxia for 50 min with or without NO (50 ppm) added to the gas mixture. Tissue loss was significantly attenuated 3 days after the insult (46).

Li et al. studied different concentrations and durations of iNO following transient MCAO for 1 h and reperfusion for 2 days. They found that the effect of iNO was dose-dependent and the maximum benefit of iNO occurred at 60 ppm for 8 and 16 h. Additionally, iNO administered for 8 h improved recovery from subarachnoid hemorrhage and reduced the inflammatory response (44).

Interestingly, one study by Zhu showed that inhaled nitric oxide provided neuroprotection only to male rats subjected to hypoxia-ischemia (47).

#### Clinical Trials

“Inhaled Nitric Oxide in Brain Injury” (NCT03260569) is an ongoing trial to evaluate the effect of 20 ppm iNO on pulmonary mechanics in adults older than 18 years following traumatic brain injury.

### Carbon Dioxide

Carbon dioxide (CO_2_) is the fourth most common gas component in air following nitrogen, oxygen, and argon. *In vivo*, CO_2_ regulates min ventilation and cerebral blood flow. In the past few decades, overwhelming evidence has linked hypocapnia in preterm neonates to an increased risk of periventricular leukomalacia and intraventricular hemorrhage (90, 91). In contrast, permissive hypercapnia in preterm neonates is safe and reduces the length of mechanical ventilation for infants, which helps decrease the incidence of bronchopulmonary dysplasia (92).

#### Preclinical Studies

Vannucci et al. studied the relationship between various concentrations of CO_2_ and hypoxic-ischemic injury. They determined that pups subjected to HI without supplemental CO_2_ had a partial pressure of CO_2_ (PCO_2_) of ~26 mmHg, and inhaling 3, 6, and 9% CO_2_ at the time of HI was associated with a PCO_2_ of 38, 55, and 71 mmHg, respectively (36). In a subsequent study, Vannucci et al. determined that extreme hypercapnia (PCO_2_ ~ 100 mmHg) can be achieved by inhaling 15% CO_2_ (37).

Based on the Rice-Vannucci model, hypocapnia in neonatal rats at the time of HI results in the most significant brain damage. Normocapnia (3% CO_2_) produced less brain damage, while hypercapnia (6% CO_2_) was associated with minimal or no brain damage based on histopathologic evaluation of cerebral hemisphere affected by hypoxic-ischemic injury via Rice-Vannucci model (36). Similarly, Kohzuki et al. found that brain injury was significantly ameliorated in neonatal rats treated with 6% CO2 during HI for 30 min and staircase test scores improved compared to controls when tested at 30 days (35). Vannucci et al. determined that mild hypercapnia (6% CO_2_) and normocapnia (3% CO_2_) at the time of HI preserved cerebral blood flow in contrast to hypocapnia (0% CO_2_), which reduced cerebral blood flow (38). Vannucci et al. found that neonatal rats exposed to extreme hypercapnia (15% CO_2_) at the time of hypoxic-ischemic injury had significantly reduced cerebral blood flow and the greatest brain infarct size compared to normocapnic littermates (37).

#### Clinical Trials

“Hypoxic-Ischemic Encephalopathy Therapy Optimization in Neonates for Better Neuroprotection with Inhalative CO_2_ (HENRIC)” is a single-center, open-label, interventional trial (NCT02700854). The study is recruiting mechanically ventilated infants undergoing therapeutic hypothermia for HIE in Hungary. The study aim is to evaluate the safety and feasibility of a low-concentration CO_2_ gas mixture (5% CO_2_ + 95% air) with the hypothesis that hyperventilation driven by metabolic acidosis harms the brain.

### Carbon Monoxide

Carbon monoxide (CO) is a colorless, odorless, and tasteless gas. It has an extremely high affinity to bind to hemoglobin and form carboxyhemoglobin. Compared to hemoglobin, carboxyhemoglobin has a significantly lower capacity to carry oxygen, which leads to cardio- and neurotoxicity (93).

#### Preclinical Studies

At high concentrations, CO is thought to be neurotoxic (29). However, lower levels of CO are neuroprotective (33). *In vitro*, CO upregulated Bcl-2 in hippocampus cells (31). In addition, CO prevents oxidative stress-induced cortical astrocyte apoptosis by preventing dissipation of mitochondrial membrane potential, caspase 3 activation, and cytochrome c release (30). At the mitochondrial level, CO prevents membrane depolarization induced by calcium and inhibits mitochondrial swelling. It also prevents membrane pore formation by modulating adenine nucleotide translocase (ANT) activity (30). ANT plays an essential rule in the stability of the mitochondrial inner membrane. It is influenced by anti- and proapoptotic agents (i.e., Bcl family) leading to inhibition or activation of pore formation in the mitochondrial membrane, respectively (94).

Electrical acupuncture into rat brains subjected to hypoxic-ischemic injury leads to increased intrinsic CO levels via overexpression of heme oxygenase 1 (HO-1) in the cortex and was associated with decreased cortical injury (34). Exogenous CO mimics the neuroprotective effects of HO-1 by reducing autoimmune neuroinflammation (95). CO causes upregulation of HO-1 by promoting cytoplasmic dissociation of Nrf2 from Keap1. This dissociation leads Nrf2 to translocate to the nucleus and bind to the antioxidant response elements (ARE) (Supplementary Figure 1) (32).

Inhalation of 125 and 250 ppm of CO for 18 h following 90 min of transient MCAO and 48 h of reperfusion was associated with smaller infarct size and reduced brain edema (33). NRF2 knockout adult mice lost CO neuroprotection following permanent MCAO (32).

#### Clinical Trials

Currently, CO for neuroprotection is not being investigated in clinical studies.

### Oxygen

Oxygen is the third most common element in the universe and is considered the cornerstone of life. Water in plants and algae produce oxygen, which mitochondria use to produce ATP. Reactive oxygen species (ROS) like superoxide are formed by a partial reduction of oxygen during cellular metabolism and are exaggerated by cellular injury such as hypoxic-ischemic injury.

In 2011, AAP changed the neonatal resuscitation program (NRP) guidelines to resuscitate term infants with room air instead of 100% based on two metanalysis studies showing less mortality in infants in room air (96, 97). This change was adopted after major therapeutic hypothermia for HIE trials were completed raising the question as whether resuscitation with supplemental oxygen may affect hypothermic neuroprotection. Dalen et al. showed that resuscitation with 100% oxygen negated the neuroprotective benefits of hypothermia in neonatal rats subjected to HI (49).

#### Preclinical Studies

The evidence regarding resuscitation with 100% oxygen is conflicting. A recent study done by Smit et al. failed to show statistical difference in cortical and hippocampal volume loss or in short-term neurological testing between immature rats resuscitated with either 21 or 100% oxygen following a modified Rice-Vannucci model of hypoxic-ischemic brain injury (50). Newborn piglets resuscitated with 100% O_2_ improved restoration of mean arterial blood pressure with no difference in amino acid build up in cerebral striatum (glutamate, taurine, and alanine) when compared to those resuscitated with 21% O_2_ (63). Reoxygenation of asphyxiated piglets with 100% O_2_ did not change cerebral perfusion but restored nitric oxide level in the cortex to a higher level compared to those resuscitated with 21% O_2_ (65, 66).

#### Clinical Studies

“Hyperbaric Oxygen Therapy Improves Outcome of Hypoxic-Ischemic Encephalopathy” (NCT02894866) is an ongoing multicenter randomized international study that is still recruiting patients. The study aim is to evaluate the safety and efficacy of hyperbaric oxygen in term infants with HIE.

## Conclusion

HIE management remains a challenge. Although therapeutic hypothermia has improved survival in infants with HIE, 7 infants needed to be treated to prevent death or major neurodevelopmental disability. Preclinical studies show that inhaled gases are a promising adjunct therapy that could improve outcomes. Of the reviewed inhaled gases and pending the results of the CoolXenon3 Trial, xenon appears to be the most promising for clinical use. Several of the other reviewed gases such as NO and C possess anti-inflammatory properties via the Nrf2 pathway and neuroprotection promise in preclinical settings. Further clinical trials should focus on proving the efficacy and safety of inhaled gases in the clinical setting.

## Author Contributions

MW and SD contributed conception and design of the study. SS, HG, and ME searched literature and organized the database. YT wrote the first draft of the manuscript. MW wrote sections of the manuscript. All authors contributed to manuscript revision, read and approved the submitted version.

### Disclosure

Mallinckrodt Pharmaceuticals provided nitric oxide gas for a preclinical study in which all authors are involved.

### Conflict of Interest

The authors declare that the research was conducted in the absence of any commercial or financial relationships that could be construed as a potential conflict of interest.

## Abbreviations

AMPA: Alpha-Amino-3-Hydroxy-5-Methyl-4-Isoxazole Propionic Acid
ARE: antioxidant response elements
Bcl-2: B-cell lymphoma 2
CO: carbon monoxide
CO_2_: carbon dioxide
GABA receptors: γ-aminobutyric acid type A receptors
HI: hypoxia-ischemia
HIE: hypoxic-ischemic encephalopathy
HO1: heme-oxygenase 1
iNO: inhaled nitrous oxide
KA: kainite
Keap1: kelch-like ECH-associated protein 1
LDH: lactate dehydrogenase
L-NAME: N(ω)-nitro-L-arginine methyl ester
MCAO: middle cerebral artery occlusion
NAA: N-acetyl aspartate
NF- κB: nuclear factor- κB
NMDA: N-methyl-D-aspartate
NO: nitric oxide
NRF2: nuclear erythroid 2-related factor 2
OGD: oxygen and glucose deprivation
pCREB: phosphorylated cAMP response element binding protein
ROS: reactive oxygen species
Tyr216-GSK-3β: Phospho-glycogen synthase kinase-3β Tyr-216.
