# Supplementary material for: Inhaled Gases for Neuroprotection of Neonates: A Review
Source: Front Pediatr. 2020 Jan 27;7:558. doi: 10.3389/fped.2019.00558 (PMC6996209; doi:10.3389/fped.2019.00558)
Supplement: Supplementary file 1 [file Data_Sheet_1.docx]

Supplementary Material

## Supplementary Figure 1: Mechanism of action of inhaled gases.


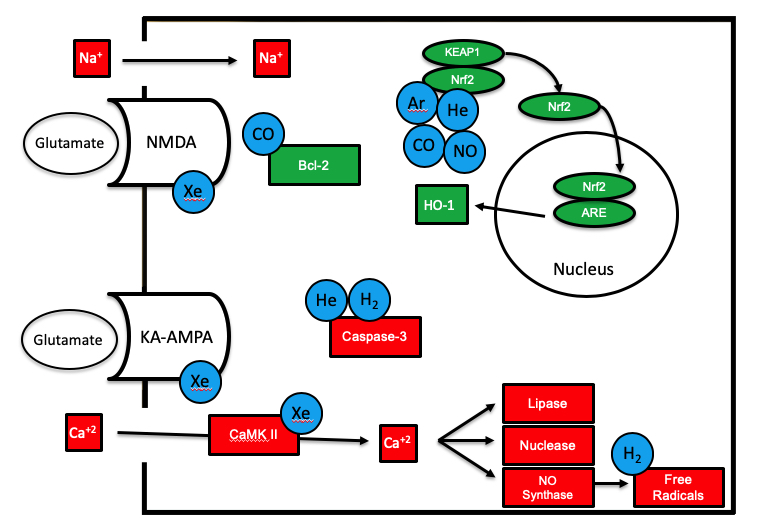


Figure legend:

During hypoxia ischemia, glutamate accumulates in the synaptic junction due to lack of ATP and binds α-amino-3-hydroxy-5-methyl-4-isoxazolepropionic acid [AMPA], kainite [KA], and N-methyl-D-aspartate [NMDA] leading to excitotoxicity. Neuroprotection occurs in several ways: Xenon [Xe] blocks NMDA and AMPA receptors and interferes with calcium/calmodulin-dependent protein kinase II [CaMKII]. Hydrogen [H_2_] and helium [He] reduce oxidative stress by inhibiting caspase-3 activity. He, extrinsic nitric oxide [NO], argon [Ar] and carbon monoxide [CO] stimulate nuclear factor erythroid 2-related factor [NRF-2] dissociation and translocation to the nucleus and transcription of cytoprotective genes. One important product is Heme-oxygenase-1 [HO-1], which degrades heme and generates antioxidant molecules. B-cell lymphoma 2 [Bcl-2] is a regulator of apoptosis and is regulated by CO.
